# A Monte Carlo Study of Dynamic Phase Transitions Observed in the Kinetic *S* = 1 Ising Model on Nonregular Lattices

**DOI:** 10.3390/e27050530

**Published:** 2025-05-16

**Authors:** Yusuf Yüksel

**Affiliations:** Physics Department, Faculty of Science, Dokuz Eylul University, Tinaztepe Campus, Izmir 35390, Turkey; yusuf.yuksel@deu.edu.tr

**Keywords:** dynamic phase transitions, Monte Carlo simulations, metamagnetic anomalies

## Abstract

In the present paper, we discuss the thermodynamic and dynamic phase transition properties of the kinetic Blume–Capel model with spin-1, defined on non-regular lattices, namely decorated simple cubic, decorated triangular, and decorated square (Lieb) lattice geometries. Benefiting from the recent results obtained for the thermodynamic phase transitions of the aforementioned lattice topologies [Azhari, M. and Yu, U., J. Stat. Mech. (2022) 033204], we explore the variation of the dynamic order parameter, dynamic scaling variance, and dynamic magnetic susceptibility as functions of the amplitude, bias, and period of the oscillating field sequence. According to the simulations, a second-order dynamic phase transition takes place at a critical field period for the systems with zero bias. A particular emphasis has also been devoted to metamagnetic anomalies emerging in the dynamic paramagnetic phase. In this regard, the generic two-peak symmetric behavior of the dynamic response functions has been found in the slow critical dynamics (i.e. dynamic paramagnetic) regime. Our results yield that the characteristics of the dynamic phase transitions observed in the kinetic Ising model on regular lattices can be extended to such non-regular lattices with a larger spin value.

## 1. Introduction

When a ferromagnetic material is subjected to a time-dependent periodic magnetic field, a dynamic phase transition (DPT) may take place between dynamic ferromagnetic and dynamic paramagnetic states depending on the competition mechanism between the intrinsic relaxation time τ of the system and the period *P* of the external magnetic field [1]. In this regard, the kinetic Ising model was introduced approximately four decades ago [2,3] as a playground for elucidating the dynamic aspects of the magnetic phase transitions in model systems. Since then, the subject attracted a considerable amount of interest both in theoretical and experimental grounds [4,5,6]. For a detailed review on the DPT in classical spin models, we refer the reader to a recent review [4]. DPT emerges as a result of a dynamic symmetry breaking phenomenon [7]. In this context, in the fast critical dynamics regime (P≪τ) time series of the magnetization (m(t)), it cannot follow the external field oscillation (h(t)). Hence, the system stays in the dynamic ferromagnetic state. On the other hand, in the slow critical dynamics regime (P≫τ), one can observe a small phase lag between m(t) and h(t) sequences, corresponding to the dynamic paramagnetic regime. In this picture, the field period is an adjustable external parameter, whereas the relaxation time can be modified as a function of the field amplitude and temperature. The point $P_{c}$ at which a dynamic phase transition emerges between dynamic ferromagnetic and dynamic paramagnetic states is called the critical period. This kind of phase transition is observed in the absence of any bias field term $h_{b}$. In the presence of non-zero $h_{b}$, the phase transition disappears and a field-polarized state becomes manifested, in which the magnetic dipole moments tend to align in the direction of the applied field.

From the perspective depicted above, it is clear that there exist a number of striking similarities between the dynamic and thermodynamic phase transitions (the latter will be abbreviated as TPT). For instance, the variation of the dynamic order parameter *Q* which allows us to distinguish between dynamic ferromagnetic and dynamic paramagnetic states as a function of the applied field period strongly resembles the longitudinal magnetization versus temperature curve of a regular ferromagnetic material. In addition, the bias field $h_{b}$ in the DPT plays the role of the longitudinal magnetic field of TPT, as long as the field sequence h(t) exhibits the half-wave anti-symmetry property [8,9]. Moreover, it has been well established that DPT and TPT exhibit the same universality class as each other, provided that the effects of any constant bias field are ruled out [10,11,12]. However, the similarities between DPT and TPT cases should be treated carefully due to the fact that the presence of $h_{b}$ may drastically affect the phase diagrams plotted in various planes. For instance, in the dynamic paramagnetic regime, the variation in the dynamic response functions with respect to $h_{b}$ exhibits an unusual symmetric double-peak behavior called ”*the metamagnetic anomalies*”, a phenomenon with no equivalence in the corresponding equilibrium ferromagnetic materials [13]. The aforementioned anomalous effect was primarily observed in experimental systems, including thin ferromagnetic Co films [14,15] and magnetic alloys [16], and it was later verified by numerical simulations using the kinetic Ising model and its extensions [17,18,19,20].

Very recently, the TPT and DPT properties of the kinetic Ising model have been studied on nonregular lattices, revealing strong similarities regarding the universality aspects of phase transitions [21,22]. In this work, we aim to extend (at least in qualitative meaning) these findings to higher-spin models and explore whether the metamagnetic anomalies are peculiar to the kinetic Ising model or represent a more general feature of kinetic spin systems. To this end, we have performed detailed Monte Carlo (MC) simulations of a two-dimensional kinetic Blume–Capel (BC) [23,24] ferromagnet on non-regular lattices, including decorated simple cubic (DSC), decorated triangular lattice (DTL), and decorated square lattice (or Lieb lattice, LL) geometries. The system is subjected to a sinusoidal external field sequence with an amplitude $h_{0}$, period *P*, and an additional bias field term $h_{b}$. Our results demonstrate that the observed phenomena are not restricted to the kinetic Ising model but exhibit characteristics that are typical of kinetic spin models more broadly. The remainder of the work is organized as follows: In Section 2, we briefly outline our model and simulation details. In Section 3, we present simulation results for TPT and DPT cases, and we study the metamagnetic anomalies on different system parameters, as well as different lattice geometries. Finally, Section 4 summarizes our findings.

## 2. Model and Simulation Details

**Figure 1:**
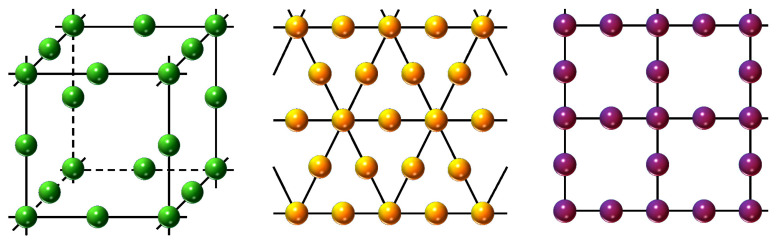
Schematic representation of the simulated lattices: DSC (**left**), DTL (**center**), and LL (**right**) in the respective order.

We simulate the magnetic properties of DSC, DTL, and LL lattices which are schematically illustrated in Figure 1, using the atomistic spin Hamiltonian given by

(1) $$H=-J\sum_{\langle i,j\rangle }S_{i}S_{j}-h\left(t\right)\sum_{i}S_{i},$$

where $S_{i}=\pm 1,\;0$ is a pseudo-spin variable, J>0 represents the ferromagnetic exchange interaction, and $h\left(t\right)=h_{0}\cos\left(\omega t\right)+h_{b}$ is a time-dependent magnetic field which is composed of an oscillating magnetic field signal and a constant bias field component $h_{b}$. The amplitude and frequency of the oscillating magnetic field are represented by $h_{0}$ and ω. The first term in Equation (1) is considered to be over the nearest-neighbor spin couples, whereas the last term is calculated over all lattice sites. Schematic representations of the simulated lattices are given in Figure 1 and they can also be found in Ref. [21]. Numerical calculations were performed using MC simulations based on the Metropolis algorithm [25] with random sweeping at each Monte Carlo step. We impose periodic boundary conditions in each direction. To calculate the critical temperatures, we examine the temperature dependence of magnetic susceptibility. Hence, in order to reduce the finite size effects, one has to consider larger lattices as much as possible. Hence, we set lateral dimension L=64 for DSC and L=324 for DTL and LL lattices. Equilibrium magnetic properties were calculated using $5\times 10^{4}$ Monte Carlo steps per site after discarding the initial 20% for thermalization. In order to reduce the statistical errors, we have also performed configurational averaging over 200 independent samples at each temperature.

We have monitored the following magnetic quantities in the absence of magnetic field:

- Spontaneous magnetization,
  (2) $$M=\frac{1}{N}\langle \sum_{i=1}^{N}S_{i}\rangle ,$$
- Longitudinal magnetic susceptibility,
  (3) $$\chi =N\left\{\langle M^{2}\rangle -{\langle M\rangle }^{2}\right\}/k_{B}T.$$

In order to investigate the dynamic phase transition properties, the lattice points have been randomly swept on a lattice with linear dimension L=48 for DSC and L=256 for DTL and LL. In order to calculate the physical quantities, $6\times 10^{3}$ period cycles of the oscillating field were considered and the initial $10^{3}$ cycles were discarded for thermalization. The period of the oscillating field is defined in terms of the Monte Carlo steps. In this regard, the length of the simulation depends on the period P=2π/ω of the periodically oscillating magnetic field. In order to guarantee that the system stays in the multi-droplet regime [26,27], we fixed the temperature at $T=0.8T_{c}$, where $T_{c}/J$ is the thermodynamic phase transition temperature of the corresponding lattice. In the absence of a bias field, 20 independent realizations have been considered to perform statistical averaging.

Using the time series of magnetization, the dynamic order parameter in the $k^{th}$ cycle can be calculated via [11]

(4) $$Q\left(k\right)=\frac{1}{\left(2t_{1/2}\right)}\int_{\left(k-1\right)\left(2t_{1/2}\right)}^{k(2t_{1/2})}m\left(t\right)dt,$$

where $t_{1/2}$ denotes the half-period (i.e., $P=2t_{1/2}$) of the oscillating magnetic field. From Equation (4), the average dynamic order parameter 〈Q〉 can be evaluated by performing the averaging procedure over successive cycles of h(t). In addition, in order to locate the critical period, we calculate the dynamic scaled variance of *Q* using

(5) $$\chi_{Q}=N\left[{\langle Q^{2}\rangle }_{L}-{\langle Q\rangle }_{L}^{2}\right].$$

Finally, we have also calculated the dynamic susceptibility with respect to the bias fied $h_{b}$

(6) $$\chi_{b}=\frac{d\langle Q\rangle }{dh_{b}}.$$

Note that we set $k_{B}=1$ and scale the field amplitudes in units of *J* for the sake of convenience in our calculations.

## 3. Results

### 3.1. TPT Properties

**Figure 2:**
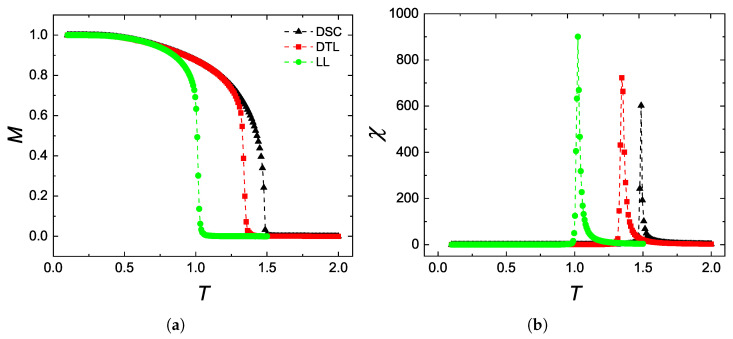
Temperature dependencies of (**a**) the magnetization and (**b**) magnetic susceptibility of a basic BC ferromagnet defined on DSC (L=64), DTL (L=324), and LL (L=324) lattices.

Before analyzing the DPT properties of the systems, one important task is to locate the transition temperature at which a ferromagnetic–paramagnetic phase transition takes place. For this aim, we have performed simulations on equilibrium systems in the absence of magnetic field effects, and calculated the longitudinal magnetization and the corresponding magnetic susceptibility for DSC, DTL and LL lattices using Equations (2) and (3). As shown in Figure 2a, each lattice structure undergoes a second order phase transition at a critical temperature $T_{c}$. Numerical value of this temperature can be closely estimated by examining the maxima of magnetic susceptibility (Figure 2b). According to the simulations, the lowest transition temperature is achieved by LL lattice due to its reduced effective coordination number $z_{eff}$. Specifically, the LL structure has an average coordination number $z_{eff}=8/3$, while for DSC and DTL lattices, we have $z_{eff}=3$ [21]. Furthermore, the transition temperature of DSC is found to be larger than that of DTL, which can be attributed to the fact that the former is a three-dimensional lattice, and MC simulations successfully distinguish between the dimensionality of lattices with the same coordination number. Additionally, the estimated transition temperature values are presented in Table 1 and can be compared with those obtained in Ref. [21] using finite-size scaling (FSS) analysis, showing good agreement. Note that the transition temperature values reported in this work correspond to the pseudo-critical temperatures since we have estimated the values by examining the magnetic susceptibility versus temperature curves. However, this fact has no significant effect on the system’s dynamic magnetic behavior.

### 3.2. DPT Properties

**Figure 3:**
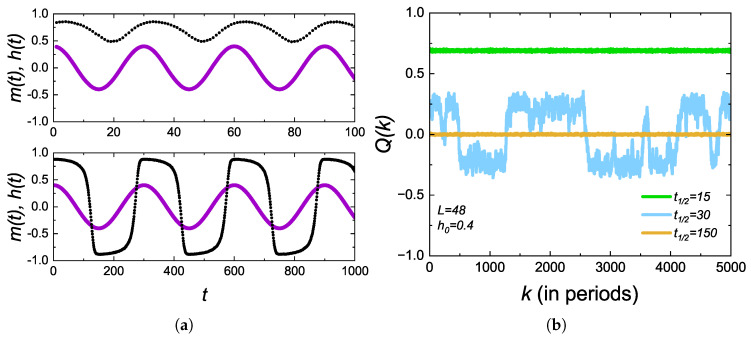
(**a**) Time series of instantaneous magnetization m(t) (dotted lines) and the field sequence h(t) (solid lines) calculated for DSC with $h_{0}=0.4$ corresponding to the fast critical (upper panel, $P=2t_{1/2}=30$) and the slow critical (lower panel, $P=2t_{1/2}=300$) dynamics regime. (**b**) Dynamic order parameter Q(k) in the $k^{th}$ cycle.

Once we have calculated the transition temperatures, the DPT properties can be investigated accordingly. In Figure 3a, we show, for DSC, the time series of instantaneous magnetization m(t) and the applied field sequence h(t) in the absence of bias. In the fast critical dynamics regime (upper panel, P=30), m(t) cannot follow the alternating external field but oscillates around a non-zero value, indicating the dynamic ferromagnetic phase, whereas for the slow critical dynamics regime (lower panel with P=300), m(t) exhibits a periodic reversal with a small delay with 〈Q〉≈0 corresponding to the dynamic paramagnetic regime. The existence of DPT can also be verified by the investigation of the dynamic order parameter Q(k) as a function of cycle index *k*, which can be calculated from Equation (4) and using the time series data shown in Figure 3a. From now on, we define the parameter half-period $t_{1/2}=P/2$ as a convention to identify the period of the external field. According to Figure 3b, below the critical period $\left(t_{1/2}<t_{1/2}^{c}\right)$, a single domain of magnetic moments is favored with Q(k)≠0, whereas for $t_{1/2}>t_{1/2}^{c}$ nucleated droplets of small spin clusters, it results in Q(k)≈0.

Around the dynamic critical point $\left(t_{1/2}\approx t_{1/2}^{c}\right)$, large fluctuations arise from the abrupt reversals of large spin clusters, leading to a DPT between dynamically ordered and disordered states. One has to keep in mind that the results shown in Figure 3 also qualitatively hold true for DTL and LL geometries.

**Figure 4:**
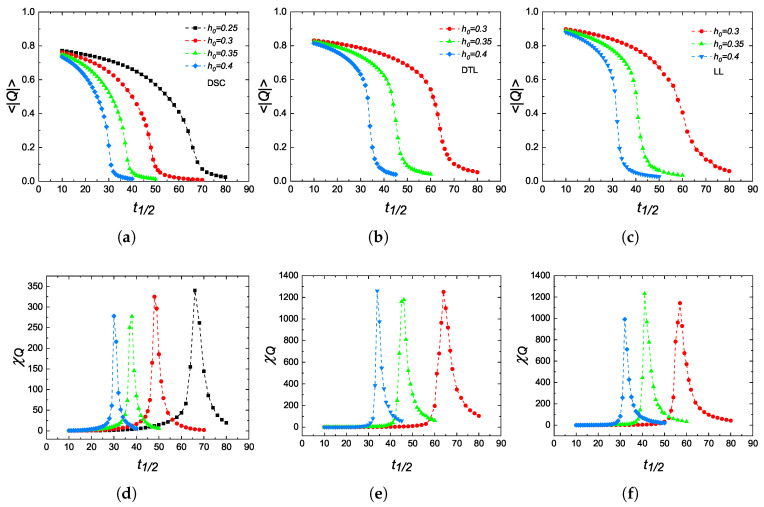
(**a**–**c**): Variations in the dynamic order parameter 〈|Q|〉 as a function of half-period $t_{1/2}$ respectively calculated for DSC, DTL, and LL geometries with some selected values of field amplitude $h_{0}$. The corresponding scaled variance $\chi_{Q}$ curves are also shown in (**d**–**f**).

An inspection of Q(k) gives a first guess about the location of the critical half-period $t_{1/2}^{c}$ of the system. In order to identify the magnetic ordering and to precisely locate the dynamic critical region, we calculate the absolute value of the average dynamic order parameter 〈|Q|〉, and the corresponding dynamic scaled variance $\chi_{Q}$ defined by Equation (5). The results are illustrated in Figure 4. It is worth emphasizing that 〈|Q|〉 continuously undergoes a dynamic phase transition independent from the lattice type being considered. Moreover, it is clear that as the field amplitude $h_{0}$ increases, the dynamic critical point estimated from the period dependencies of $\chi_{Q}$ curves shifts to smaller periods as the system’s relaxation time becomes comparable to the field period, allowing the magnetization to follow the alternating field sequence more easily with the energy gain supplied by the magnetic field. Regarding the similarities between the DPT and TPT, it can be deduced that the curves of 〈|Q|〉 and $\chi_{Q}$ versus $t_{1/2}$ in DPT play the role of spontaneous magnetization and magnetic susceptibility versus temperature curves in TPT, respectively. These findings are in good agreement with those reported for the BC model defined on a regular square lattice [28]. As a limitation of the present study, it is also important to emphasize that performing FSS calculations to extract the critical exponent ratios β/ν and γ/ν corresponding to the dynamic order parameter and the scaled variance would ensure a quantitative improvement on the reliability of the results. However, this would demand significantly large computational resources, and such analysis are out of scope in the present work.

### 3.3. Metamagnetic Anomalies

**Figure 5:**
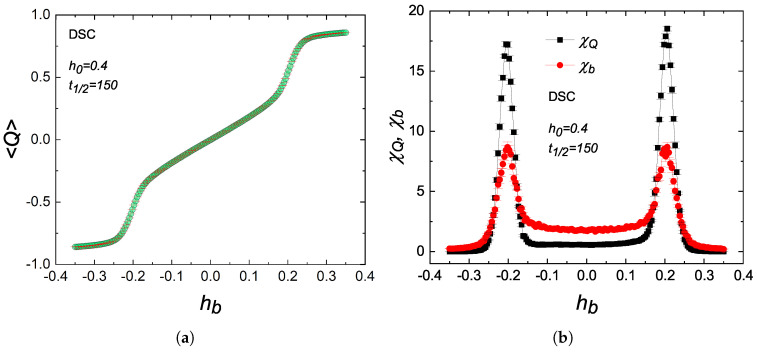
Variations in (**a**) the average dynamic order parameter 〈Q〉 and (**b**) scaled variance $\chi_{Q}$ and dynamic susceptibility $\chi_{b}$ as functions of bias $h_{b}$, obtained for a DSC lattice with L=12.

**Figure 6:**
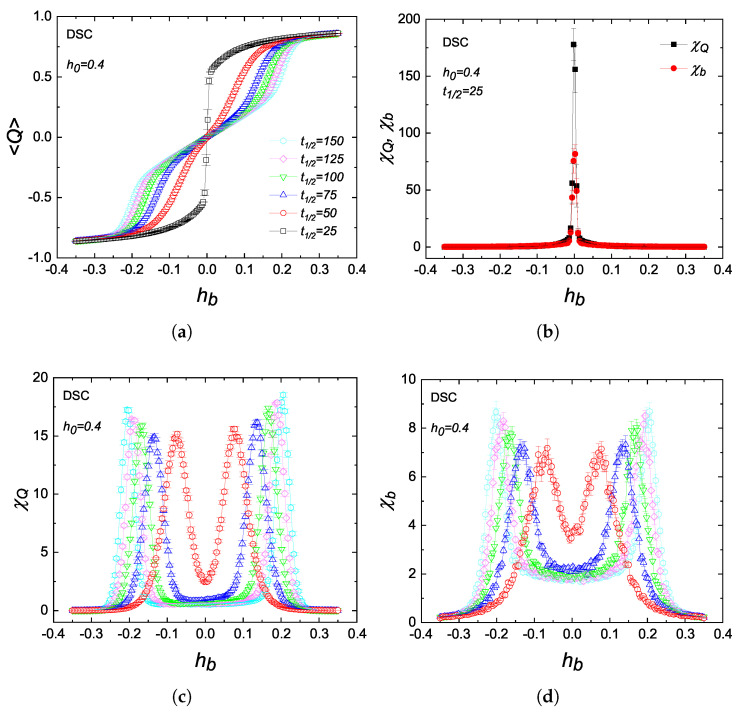
Variations in the metamagnetic behavior of a DSC lattice with some selected values of the half-period for $h_{0}=0.4$. (**a**) 〈Q〉 versus $h_{b}$. (**b**) $\chi_{Q}$ and $\chi_{b}$ against $h_{b}$ for $t_{1/2}<t_{1/2}^{c}$. (**c**) $\chi_{Q}$ and (**d**) $\chi_{b}$ versus $h_{b}$ for a variety of half-period lengths.

**Figure 7:**
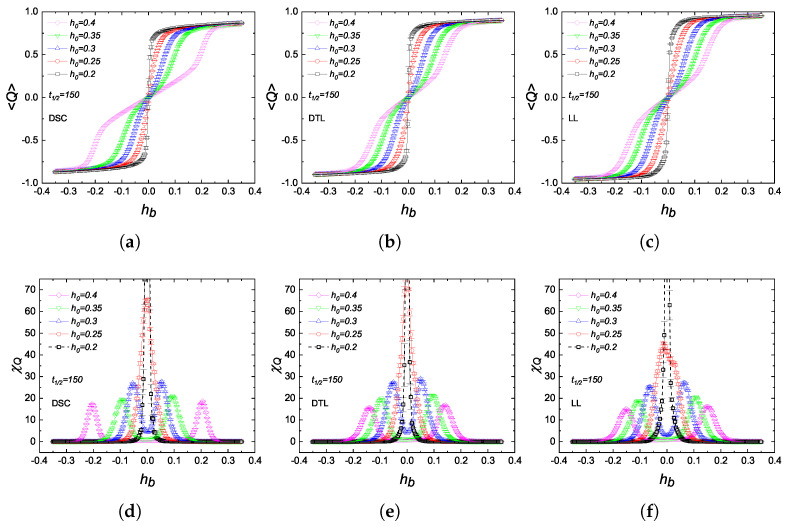
Variations in (**a**–**c**) the dynamic order parameter 〈Q〉 and (**d**–**f**) dynamic scaling variance $\chi_{Q}$ as functions of the bias field $h_{b}$ calculated for the DSC, DTL, and LL lattices, respectively. Each curve corresponds to a particular field amplitude $h_{0}$. The half-period is fixed as $t_{1/2}=150\gg t_{1/2}^{c}$.

As an example of a dissimilarity between DPT and TPT cases, the results of a recent experimental study are particularly noteworthy. In that work, Berger and coworkers [14] observed anomalous behavior in the dynamic susceptibility and the scaled variance of Co-based thin films, both plotted as functions of the applied bias field in the dynamic paramagnetic regime. Supported by subsequent numerical studies [17,18,19,20], this behavior is manifested as two symmetric peaks with respect to zero bias, which is referred to as “*metamagnetic anomalies*” or “*side bands*” [14]. This kind of behavior has no analogy in equilibrium phase transitions [13]. Figure 5 shows such kinds of behavior observed in the dynamic critical region $\left(P>P_{c}\right)$ in the phase space of a DSC lattice. The quantities demonstrated in Figure 5 are not significantly affected by the system size [18]. Hence, in order to reduce the computational time, we set L=12 for DSC and L=32 for DTL and LL lattices in the following discussions. In addition, we also consider sample averaging over 10 independent samples to reduce the statistical errors. The error bars in Figure 5, Figure 6 and Figure 7 were calculated using the Jackknife method [25]. A comparison of Figure 5a,b yields that the symmetric peaks emerge when 〈Q〉 varies relatively in a steep fashion in the vicinity of two regions denoted as $h_{b}\approx \pm h_{b}^{peak}$. The linear trend in 〈Q〉 around $h_{b}=0$ results in a zero slope, and consequently, one observes a wide minimum in scaled variance $\chi_{Q}$ and dynamic susceptibility $\chi_{b}$ for $h_{b}<\left|h_{b}^{peak}\right|$. These observations are also valid for DTL and LL lattices. Therefore, these metamagnetic anomalies are not restricted to kinetic spin models defined on regular lattices but can also be generalized to nonregular lattices and kinetic models with arbitrary spin.

In Figure 6, we discuss the half-period dependence of the metamagnetic anomalies on a DSC lattice. As reflected in Figure 6a, for $t_{1/2}<t_{1/2}^{c}$, 〈Q〉 exhibits a discontinuous jump resulting in a narrow peak behavior in $\chi_{Q}$ and $\chi_{b}$ at $h_{b}=0$. Note that dynamic critical point is located at $t_{1/2}^{c}=31$ for the DSC lattice when $h_{0}=0.4$. As the period increases, the curves become smoother, and the divergent single peak evolves into symmetric double-peak behavior, indicating a dynamic phase transition between dynamically ordered and disordered phases. Furthermore, the peaks spread out with increasing period. We also observe that $\chi_{Q}$ has a larger value than $\chi_{b}$. These observations are in qualitative agreement with recent studies on the kinetic BC model on a regular square lattice [18], except for the fact that our $\chi_{Q}$ data do not show any local minimum at $h_{b}=0$.

Last but not least, we investigate the dependence of metamagnetic anomalies on the field amplitude and lattice topology for a fixed half-period, $t_{1/2}=150$, which is extremely large compared to the critical half-period of the individual lattices. As shown in Figure 7, the lattice topology does not have a significant effect on the sidebands. Hence, the qualitative behavior remains identical to that observed in the regular lattice kinetic BC model [18]. On the other hand, as $h_{0}$ increases, the $\left(\langle Q\rangle ,h_{b}\right)$ curves exhibit a middle plateau, indicating that the $\chi_{Q},\chi_{b}$ versus $h_{b}$ curves evolve from a single narrow peak to symmetric and finite double-peak structure. Moreover, the $|h_{b}^{peak}|$ value tends to extend towards larger values due to the competition between $h_{0}$ and $h_{b}$. Namely, larger field amplitudes require a stronger bias field as a result of the enhanced dynamic paramagnetism. These observations are in accordance with those reported for the kinetic Ising and BC models [7,14,18].

## 4. Conclusions

In conclusion, we have investigated the thermodynamic and dynamic phase transition properties of a kinetic Blume–Capel model on non-regular lattices, namely, DSC, DTL, and LL. In the absence of a magnetic field, evaluating the spontaneous magnetization and magnetic susceptibility versus temperature curves yields that the systems undergo a second-order phase transition with Curie points matching well with those obtained from finite-size scaling analysis. After evaluating the transition temperatures, we have focused our attention on the dynamic aspects of the phase transitions in the vicinity of multi-droplet regime $\left(T=0.8T_{c}\right)$. In case of zero bias, the DPT is of second order. As a general outcome, by comparing the magnetization versus temperature curves of TPT (Figure 2) with dynamic order parameter versus the half-period curves of DPT (Figure 4) with each other, we have qualitatively found that the external field period *P* in DPT plays the role of the temperature *T* in TPT. In the last part, we have investigated the metamagnetic anomalies with varying amplitude, period, and lattice geometries. According to obtained results, these metamagnetic anomalies, as well as DPT characteristics are not restricted to kinetic spin models defined on regular lattices but can also be generalized to nonregular lattices and kinetic models with arbitrary spin. All observations are in accordance with those previously reported for the kinetic Ising model and also for its extensions.

Regarding the DPT properties, the reliability of the qualitative research conducted in the present work can be improved by calculating the dynamic critical exponent ratios corresponding to the dynamic order parameter and dynamic scaled variance. Hence, future research related to the present work could involve the universality aspects of dynamic phase transitions on the aforementioned lattice types by taking into account the effect of single-ion anisotropy energy. We hope that our results will contribute to a deeper understanding of dynamic phase transitions in kinetic spin models.

## Figures and Tables

**Table 1:** Obtained transition temperature values for the equilibrium ferromagnetic systems. The results of Ref. [21] are also given for direct comparison.

Lattice	$T_{c}$ Ref. [21]	$T_{c}^{*}$ (Present Work)
DTL	1.340(2)	1.344
LL	1.017(1)	1.024
DSC	1.483(2)	1.487

## Data Availability

The raw data supporting the conclusions of this article will be made available by the authors on request.
